# Supplementary material for: ATR inhibition using gartisertib enhances cell death and synergises with temozolomide and radiation in patient-derived glioblastoma cell lines
Source: Oncotarget. 2024 Jan 16;15:1–18. doi: 10.18632/oncotarget.28551 (PMC10791076; doi:10.18632/oncotarget.28551)
Supplement: Supplementary file 2 [file oncotarget-15-28551-s002.pdf]

Supplementary Table 2: Parametric synergy analysis using MuSyC and BRAID models to assess the relationship between gartisertib, TMZ and RT within glioblastoma cell lines

	MuSyC analysis													BRAID analysis		
	Efficacy [a]				→ TMZ potency [b]			→ RT potency [c]			→ Gartisertib potency [d]			Kappa		
**Cell line**	**TMZ + RT**	**Gartisertib + TMZ**	**Gartisertib + RT**	**Gartisertib + TMZ + RT**	**RT**	**Gartisertib**	**Gartisertib+RT**	**TMZ**	**Gartisertib**	**Gartisertib+TMZ**	**TMZ**	**RT**	**TMZ+RT**	**TMZ + RT**	**Gartisertib + TMZ**	**Gartisertib + RT**
**HW1**	Ant	Ant	Ant	Ant	Ant	Ant	Syn	Ant	Syn	Ant	Syn	Ant	Ant	Ant	Syn	Ant
**FPW1**	Ant	Ant	Ant	Syn	Syn	Syn	Syn	Ant	Syn	Syn	Syn	Syn	Ant	Ant	Syn	Syn
**JK2**	Ant	Ant	Syn	Ant	Ant	Ant	Syn	Ant	Syn	Ant	Syn	Syn	Syn	Ant	Syn	Syn
**BAH1**	Ant	Ant	Ant	Ant	Ant	Ant	Ant	Ant	Ant	Ant	Ant	Ant	Ant	Ant	Syn	Ant
**RKI1**	Ant	Ant	Ant	Ant	Ant	Syn	Ant	Syn	Ant	Syn	Syn	Syn	Syn	Ant	Syn	Syn
**MMK1**	Ant	Ant	Ant	Ant	Syn	Syn	Ant	Ant	Ant	Syn	Syn	Ant	Syn	Ant	Syn	Syn
**SJH1**	Syn	Ant	Syn	Ant	Ant	Syn	Ant	Ant	Syn	Ant	Syn	Syn	Ant	Ant	Syn	Ant
**WK1**	Ant	Ant	Ant	Ant	Ant	Syn	Syn	Ant	Syn	Syn	Syn	Ant	Ant	Ant	Syn	Syn
**RN1**	Ant	Syn	Syn	Syn	Syn	Syn	Ant	Syn	Syn	Syn	Syn	Syn	Syn	Ant	Syn	Syn
**SB2b**	Ant	Ant	Ant	Syn	Ant	Ant	Ant	Ant	Ant	Ant	Syn	Ant	Ant	Ant	Syn	Ant
**MN1**	Ant	Ant	Ant	Ant	Ant	Syn	Ant	Ant	Ant	Ant	Syn	Ant	Syn	Ant	Ant	Ant
**PB1**	Ant	Ant	Ant	Syn	Ant	Syn	Ant	Syn	Ant	Ant	Ant	Ant	Ant	Ant	Syn	Syn
**Total (syn)**	1	1	3	4	3	8	4	3	6	5	10	5	5	0	11	7
**Total (ant)**	11	11	9	8	9	4	8	9	6	7	2	7	7	12	1	5

[a] synergistic efficacy (β) of the below drug combination

[b-d] whether the below drug/drug combination enhances the potency (synergistic) of TMZ, RT or gartisertib or does not enhance potency (antagonistic).

Abbreviations: antagonism (ant), synergy (syn), temozolomide (TMZ), RT (radiation)
